# Pathophysiology of the Effects of Alcohol Abuse on the Endocrine System

**DOI:** 10.35946/arcr.v38.2.08

**Published:** 2017

**Authors:** Nadia Rachdaoui, Dipak K. Sarkar

**Affiliations:** Nadia Rachdaoui, Ph.D., is an Assistant Research Professor, and Dipak K. Sarkar, Ph.D., D.Phil., is Board of Governors Distinguished Professor, in the Rutgers Endocrine Research Program, Department of Animal Sciences, Rutgers University, New Brunswick, New Jersey

**Keywords:** Alcohol consumption, alcohol use, abuse, and dependence, harmful effects of alcohol, pathophysiology, endocrine system, hypothalamus, pituitary gland, hormones, hormonal disturbances, endocrine pancreas, endocrine adipose tissue, immune system, humans, animal models

## Abstract

Alcohol can permeate virtually every organ and tissue in the body, resulting in tissue injury and organ dysfunction. Considerable evidence indicates that alcohol abuse results in clinical abnormalities of one of the body’s most important systems, the endocrine system. This system ensures proper communication between various organs, also interfacing with the immune and nervous systems, and is essential for maintaining a constant internal environment. The endocrine system includes the hypothalamic–pituitary–adrenal axis, the hypothalamic–pituitary–gonadal axis, the hypothalamic–pituitary–thyroid axis, the hypothalamic–pituitary–growth hormone/insulin-like growth factor-1 axis, and the hypothalamic–posterior pituitary axis, as well as other sources of hormones, such as the endocrine pancreas and endocrine adipose tissue. Alcohol abuse disrupts all of these systems and causes hormonal disturbances that may result in various disorders, such as stress intolerance, reproductive dysfunction, thyroid problems, immune abnormalities, and psychological and behavioral disorders. Studies in both humans and animal models have helped shed light on alcohol’s effects on various components of the endocrine system and their consequences.

Alcohol abuse can result in clinical abnormalities of one of the body’s most important systems, the endocrine system. Together with the nervous system, the endocrine system is essential for controlling the flow of information between the different organs and cells of the body. The nervous system is responsible for rapid transmission of information between different body regions, whereas the endocrine system, which is composed of a complex system of glands that produce and secrete hormones directly into the blood circulation, has longer-lasting actions. Together, the nervous system and the endocrine system ensure proper communication between various organs of the body to maintain a constant internal environment, also called homeostasis. Almost every organ and cell in the body is affected by the endocrine system. Its hormones control metabolism and energy levels, electrolyte balance, growth and development, and reproduction. The endocrine system also is essential in enabling the body to respond to, and appropriately cope with, changes in the internal or external environments (e.g., changes in the body’s temperature or in the electrolyte composition of the body’s fluids) as well as to respond to stress and injury.

Both acute and chronic exposure to alcohol may have differential direct and indirect effects on endocrine functions. Alcohol intoxication induces hormonal disturbances that can disrupt the body’s ability to maintain homeostasis and eventually can result in various disorders, such as cardiovascular diseases, reproductive deficits, immune dysfunction, certain cancers, bone disease, and psychological and behavioral disorders. Alcohol use has been shown to affect many hormone systems, including the hypothalamic–pituitary–adrenal (HPA) axis, the hypothalamic– pituitary–gonadal (HPG) axis, the hypothalamic–pituitary–thyroid (HPT) axis, the hypothalamic–pituitary–growth hormone/insulin-like growth factor-1 (GH/IGF-1) axis, and the hypothalamic–posterior pituitary (HPP) axis. After a brief overview of the hormones of the hypothalamus and pituitary gland, this article discusses the adverse effects of both acute and chronic alcohol exposure on the different components of these hormone systems based on recent findings from human and animal studies. In addition, alcohol influences the release and actions of the pituitary hormone prolactin (outlined in the sidebar “Alcohol and Prolactin”) as well as of hormones produced and released in other tissues, such as the endocrine pancreas and the adipose tissue (reviewed in the sidebar “Alcohol and Other Endocrine Tissues”).

## Hormones of the Hypothalamus and Pituitary Gland

The hypothalamic–pituitary axis can be considered the coordinating center of the endocrine system. The hypothalamus is the main neural control center, also known as the “master switchboard,” which coordinates nervous and endocrine system functions. The hypothalamus consolidates inputs derived from higher brain centers, various environmental cues, and endocrine feedback. Neurons within the hypothalamus produce and secrete releasing hormones, such as corticotropin-releasing factor (CRF), luteinizing hormone–releasing hormone (LHRH), thyrotropin-releasing hormone (TRH), and growth hormone–releasing hormone (GRH), as well as inhibiting hormones, such as somatostatin and dopamine, directly into the blood vessel connecting the hypothalamus with the pituitary gland (i.e., the hypothalamic– hypophyseal portal vein). These hormones then control the synthesis and release of hormones in the pituitary gland. The pituitary gland comprises two sections—the adenohypophysis, or anterior lobe, and the neurohypophysis, or posterior lobe. In response to signals from the hypothalamus, the anterior pituitary produces and secretes trophic hormones, which are hormones that have a growth effect on the organs or tissues they are targeting. They include, among others, adrenocorticotropic hormone (ACTH), thyroid-stimulating hormone (TSH), follicle-stimulating hormone (FSH), luteinizing hormone (LH), prolactin, and growth hormone (GH) and modulate the functions of several peripheral endocrine glands (i.e., adrenal glands, thyroid, and gonads) and tissues (e.g., breast, muscle, liver, bone, and skin) (see the table).

The posterior or neurohypophyseal lobe of the pituitary contains the terminals of certain neurons (i.e., magnocellular vasopressin- and oxytocin-producing neurons) originating in two specific sections (i.e., the paraventricular nuclei [PVN] and supraoptic nuclei) of the hypothalamus. These neurons secrete primarily two hormones from the posterior pituitary into the systemic blood: arginine vasopressin (AVP), which controls the renal water handling and cardiovascular functions, and oxytocin, which regulates milk ejection during lactation and uterine contractions during birth. Evidence also indicates that both AVP and oxytocin act not only as hormones but also as neuromodulators and neurotransmitters within the central nervous system (de Wied et al. 1993; Stoop 2014). However, AVP and oxytocin also can be produced in another group of neurons in the PVN and supraoptic nuclei (i.e., in the parvocellular neurons) and released into the hypothalamic–hypophyseal portal vessels to reach the anterior pituitary. There, AVP acts synergistically with CRF to promote secretion of ACTH (Plotsky 1991). In contrast, oxytocin acts on specialized cells in the anterior pituitary to promote prolactin secretion (Sarkar and Gibbs 1984).

## Alcohol and the HPA Axis

### Normal Functioning of the HPA Axis

**Figure 1:**
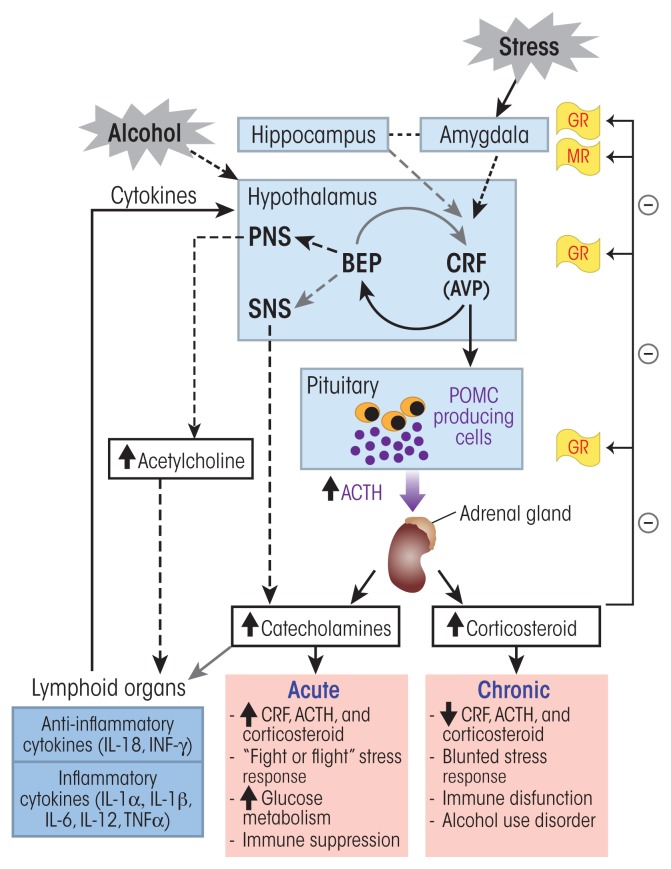
Alcohol’s effects on the hypothalamic–pituitary–adrenal (HPA) axis and the stress response. Alcohol can stimulate neurons in the paraventricular nucleus (PVN) of the hypothalamus to release corticotropin-releasing factor (CRF) and arginine vasopressin (AVP). Stress sensed in the amygdala also elicits a similar activation of this stress response pathway. In the anterior pituitary, CRF stimulates the production of proopiomelanocortin (POMC), which serves as the prohormone for adrenocorticotropic hormone (ACTH). AVP potentiates the effects of CRF on ACTH release from the anterior pituitary. ACTH stimulates cells of the cortical portion of adrenal glands to produce and release glucocorticoid hormones (i.e., cortisol). High levels of glucocorticoids inhibit CRF and ACTH release through a negative feedback by binding to glucocortiocoid receptors (GRs) and mineralocorticoid receptors (MRs) in various brain regions. Neurons in the arcuate nucleus of the hypothalamus release β-endorphin (BEP), which also regulates CRF release. BEP also acts on the autonomous nervous system and inhibits the sympathetic nervous system (SNS) stress response. CRF, ACTH, and glucocorticoids also act on different organs of the immune system and stimulate cytokine production and release into the general circulation. These cytokines then reach the brain where they trigger a neuroimmune response that sensitizes the stress-response pathway. Acute exposure to alcohol stimulates the HPA-axis stress response and induces suppression of cytokine production. In contrast, chronic exposure to alcohol induces a blunted HPA-axis stress response characterized by an absence of negative feedback control of this pathway and an increase in proinflammatory cytokines, such as interleukin-6 (IL-6) and tumor necrosis factor alpha (TNFα), leading to stress intolerance, immune dysfunction and alcohol use disorder.

The HPA axis (figure 1) is one of the endocrine pathways most sensitive to the effects of alcohol abuse. This hormone system controls the stress-response pathways and regulates many of the body’s physiological processes, such as metabolic, cardiovascular, and immune functions. It integrates physical and psychosocial stimuli to allow the body to maintain homeostasis. In response to stress (i.e., psychological, physical, or infectious stressors) or other homeostatic challenges, neurons in the PVN of the hypothalamus synthesize and secrete CRF and AVP. At the anterior pituitary, CRF binds to CRF1 receptors and stimulates specific cells (i.e., corticotropic cells) to synthesize and secrete a peptide called proopiomelanocortin (POMC). POMC can be cleaved into several smaller peptides, including ACTH; β-endorphin (BEP); and three similar peptides called α-, β-, and γ-melanocyte stimulating hormones. The POMC in the anterior pituitary primarily is processed into ACTH, whereas BEP mainly is derived from POMC produced in the hypothalamus (i.e., the ventromedial arcuate nucleus). At the same time, the AVP binds to V1b receptors, potentiating the effects of CRF on ACTH production in the anterior pituitary.

ACTH then is released into the systemic circulation, where it binds to specific receptors (i.e., melanocortin type 2 receptors) on cells in an area called the zona fasciculata in the outer layer (i.e., cortex) of the adrenal glands that are located on top of the kidneys. There, ACTH stimulates the production of glucocorticoid hormones—mainly cortisol in humans and corticosterone in rodents. These hormones then initiate a cascade of biological responses that help counteract the altered homeostatic state. Glucocorticoids achieve their effects by binding to widely distributed high-affinity mineralocorticoid receptors and low-affinity glucocorticoid receptors on their target cells. These receptors then translocate to the cell nucleus, where they bind to specific DNA sequences called glucocorticoid response elements of genes that are responsive to glucocorticoids, thereby positively or negatively regulating the expression of those genes.

The activity of the HPA axis is regulated through several feedback mechanisms. The principal protection against overactivation of the HPA axis involves the glucocorticoids (e.g., cortisol) through a negative feedback loop. Thus, glucocorticoids bind to mineralocorticoid (type 1) receptors and glucocorticoid (type 2) receptors in the hypothalamus, hippocampus, and pituitary. This binding decreases CRF, AVP, and ACTH production (figure 1). An additional negative feedback mechanism involves the BEP produced from POMC, which is synthesized in the ventromedial arcuate nucleus of the hypothalamus after stress activation. CRF release by cells from the PVN of the hypothalamus activates this BEP synthesis and release, which then inhibits further CRF release, creating a negative feedback cycle (Plotsky et al. 1991). These feedback processes help to maintain the cortisol concentration within a narrow physiological window and switch off the stress response (Myers et al. 2012; Wynne and Sarkar 2013).

**Alcohol and Prolactin**

Prolactin, also known as luteotropin, is a polypeptide hormone produced and secreted by specialized cells in the anterior pituitary called lactotropes. As the name indicates, prolactin is involved in the maintenance of lactation by the mammary glands. However, prolactin also has been implicated in a plethora of other biological functions or responses, such as mammary-gland development; reproduction; immune functions; and behavioral functions, including learning, memory, and adaptation. Prolactin is regulated by numerous mechanisms, including both inhibitory and stimulatory signals from the hypothalamus. The main hypothalamic factor responsible for inhibition of prolactin release is dopamine. Thus, prolactin secretion is controlled by a short-loop inhibitory feedback effect, whereby elevated prolactin levels in the circulation stimulate the hypothalamus to release dopamine, which then acts on the pituitary to stop further prolactin release. Dopamine also can block prolactin release directly at the level of lactotropes. In addition to dopamine, γ-aminobutyric acid released by hypothalamic neurons inhibits prolactin release. Conversely, several hypothalamic factors stimulate prolactin release from the anterior pituitary, including thyrotropin-releasing hormone, vasoactive intestinal peptide, oxytocin, β-endorphin, neurotensin, substance P, serotonin, and prostaglandins.

Several reports have indicated that chronic alcohol use can cause excessive levels of prolactin in the blood (i.e., hyperprolactinemia) in both men and women. For example, persistent hyperprolactinemia was observed in women with alcohol use disorder (AUD) and no clinical evidence of alcoholic liver cirrhosis who reported an average daily alcohol intake of 170 g (i.e., approximately 12 standard drinks) for 2 to 16 years (Valimaki et al. 1984). Elevated prolactin levels also were reported in women with AUD and admitted for alcoholism treatment who reported drinking an average of 84 g of alcohol (i.e., approximately 7 standard drinks) per day for at least 7 years (Seki et al. 1991). Alcohol-induced hyperprolactinemia also was evident in postmenopausal women (Gavaler 1994) and in men with AUD (Soyka et al. 1991).

Studies in nonhuman primates and laboratory animals have confirmed an alcohol-induced hyperprolactinemia. For example, acute ethanol administration increased serum prolactin levels in male (Seilicovich et al. 1985) and female (Dees and Kozlowski 1984) rats. Similarly, chronic self-administration of alcohol (3.4 g/kg/day) in female monkeys was associated with an increase in plasma prolactin levels (Mello et al. 1988) as well as apparent enlargement (i.e., hyperplasia) of the pituitary as demonstrated by immunocytochemical examination (Mello et al. 1983). Ethanol also increased plasma prolactin levels and pituitary weight both in female rats with normal menstrual cycles and in rats whose ovaries had been removed (i.e., ovariectomized rats) and promoted estradiol-induced development of prolactin-producing benign tumors (i.e., prolactinomas) in the pituitary (De et al. 1995). Finally, ethanol increased basal and estradiol-mediated proliferation of lactotropic cells in primary cultures of mixed anterior pituitary cells, but failed to do so in cultures of only lactotropic cells, indicating that ethanol’s effects on proliferation require cell-to-cell communication between lactotropic and other pituitary cells (De et al. 2002).

The inhibitory action of hypothalamic dopamine on pituitary prolactin secretion is mediated by the dopamine G-protein–coupled D2 receptors (D2R), which interact with regulatory molecules called G-proteins and specifically a subtype called adenylyl-cyclase–inhibitory Gi/Go (Ben-Jonathan et al. 2001; Sarkar 2010). There are two isoforms of the D2R, a long (D2L) and a short (D2S) isoform.^1^ Chronic exposure to ethanol increases the expression of prolactin mRNA and of D2L mRNA but decreases expression of D2S both in the pituitary of Fischer-344 rats and in primary cultures of anterior pituitary cells (Oomizu et al. 2003). In addition, exposure of ovariectomized rats to ethanol for 2 to 4 weeks reduced the expression of two other G-proteins, Gi2 and Gi3 (Chaturvedi and Sarkar 2008). Similar results were found in experiments using various cell culture models (Sengupta and Sarkar 2012).

Finally, ethanol treatment had differential effects on various G-proteins in cells expressing only D2S or D2L, eliciting a marked increase in Gs expression and a decrease in Gi3 expression in D2S cells but a moderate increase in Gs and marked increase in Gi3 expression in D2L (Sengupta and Sarkar 2012). Taken together these studies indicate that ethanol diminishes dopamine’s ability to inhibit prolactin secretion by altering the processing (i.e., splicing) of D2R mRNA, promoting the increase of the D2L isoform, as well as by differentially altering the expression of various Gi and Gs proteins in lactotropic cells.

Ethanol exposure affects prolactin production not only in adults but also in the developing fetus. Fetal alcohol exposure from day 7 to day 21 of gestation increased pituitary weight, pituitary prolactin mRNA and protein content, and prolactin plasma levels in female rats compared with control animals (Gangisetty et al. 2015). These changes are associated with decreased D2R mRNA and protein. This decrease seems to be related to reduced activity of the gene resulting from epigenetic modifications of the D2R gene. Thus, fetal ethanol exposure increased methylation of a regulatory element (i.e., the promoter) of the D2R gene, thereby reducing transcription. In addition, ethanol exposure increased the mRNA levels for several methylating enzymes and enzymes called histone deacetylases that modify the proteins (i.e., histones) around which the DNA is wound, which also interfere with transcription (Gangisetty et al. 2015). The role of these processes in ethanol-induced modifications of prolactin levels was confirmed by the finding that treatment with agents that prevent DNA methylation and/or histone deacetylase activity normalized D2R mRNA expression, pituitary weight, and plasma prolactin levels in fetal alcohol–exposed rats (Gangisetty et al. 2015).

Ethanol affects prolactin levels not only through its impact on D2R but also through changes in the production and secretion of growth factors in the pituitary that help control lactotropic cell proliferation. Specifically, ethanol exposure of ovariectomized rats for 2 to 4 weeks decreased the levels of growth-inhibitory molecules (e.g., transforming growth factor beta-1 [TGFβ-1]) and increased the levels of growth-stimulatory factors, such as TGFβ-3 and basic fibroblast growth factor, in the pituitary gland; similar results were found in isolated cell cultures enriched for lactotropes and exposed to ethanol for 24 hours (Sarkar and Boyadjieva 2007).

These and other studies (Gavaler 1994; Mello et al. 1989; Seki et al. 1991; Valimaki et al. 1984) clearly have demonstrated that chronic alcohol consumption is a positive risk factor for the development of prolactinomas and hyperprolactinemia. Common manifestations of hyperprolactinemia in women include lack of menstrual cycles (i.e., amenorrhea) and excessive or spontaneous secretion of milk (i.e., galactorrhea). Men with hyperprolactinemia typically show hypogonadism, with decreased sex drive, reduced sperm production, and impotence, and may also exhibit breast enlargement (i.e., gynecomastia), although they very rarely produce milk.

1The D2S isoform results from an exclusion of the sixth exon of the D2R gene in the mature transcript.

ReferencesBen-JonathanNHnaskoRDopamine as a prolactin (PRL) inhibitorEndocrine Reviews22672476320011173932910.1210/edrv.22.6.0451ChaturvediKSarkarDKAlteration in G proteins and prolactin levels in pituitary after ethanol and estrogen treatmentAlcoholism: Clinical and Experimental Research32580681320081833663010.1111/j.1530-0277.2008.00638.xPMC2869483DeABoyadjievaNOomizuSSarkarDKEthanol induces hyperprolactinemia by increasing prolactin release and lactotrope growth in female ratsAlcoholism: Clinical and Experimental Research2691420142920021235193810.1097/01.ALC.0000030621.35354.E0DeABoyadjievaNPastorcicMSarkarDPotentiation of the mitogenic effect of estrogen on the pituitary-gland by alcohol-consumptionInternational Journal of Oncology7364364819952155288510.3892/ijo.7.3.643DeesWLKozlowskiGPDifferential effects of ethanol on luteinizing hormone, follicle stimulating hormone and prolactin secretion in the female ratAlcohol164294331984644306910.1016/0741-8329(84)90017-xGangisettyOWynneOJabbarSFetal alcohol exposure reduces dopamine receptor D2 and increases pituitary weight and prolactin production via epigenetic mechanismsPLoS One1010e014069920152650989310.1371/journal.pone.0140699PMC4624904GavalerJSAging and alcohol: The hormonal status of postmenopausal womenSarkarDKBarnesCReproductive Neuroendocrinology of Aging and Drug AbuseBoca Raton, FLCRC Press1994365378MelloNKBreeMPMendelsonJHAlcohol self-administration disrupts reproductive function in female macaque monkeysScience22146116776791983686773910.1126/science.6867739MelloNKMendelsonJHTeohSKNeuroendocrine consequences of alcohol abuse in womenAnnals of the New York Academy of Sciences5622112401989266285910.1111/j.1749-6632.1989.tb21020.xMelloNKMendelsonJHBreeMPSkupnyAAlcohol effects on naloxone-stimulated luteinizing hormone, follicle-stimulating hormone and prolactin plasma levels in female rhesus monkeysJournal of Pharmacology and Experimental Therapeutics245389590419883133465OomizuSBoyadjievaNSarkarDKEthanol and estradiol modulate alternative splicing of dopamine D2 receptor messenger RNA and abolish the inhibitory action of bromocriptine on prolactin release from the pituitary glandAlcoholism: Clinical and Experimental Research27697598020031282481910.1097/01.ALC.0000071743.57855.BEPMC2869286SarkarDKHyperprolactinemia following chronic alcohol administrationFrontiers of Hormone Research38324120102061649310.1159/000318492SarkarDKBoyadjievaNIEthanol alters production and secretion of estrogen-regulated growth factors that control prolactin-secreting tumors in the pituitaryAlcoholism: Clinical and Experimental Research31122101210520071803469910.1111/j.1530-0277.2007.00539.xPMC2895402SeilicovichARubioMDuvilanskiBInhibition by naloxone of the rise in hypothalamic dopamine and serum prolactin induced by ethanolPsychopharmacology (Berlin)8744614631985300180910.1007/BF00432513SekiMYoshidaKOkamuraYA study on hyperprolactinemia in female patients with alcoholics[Article in Japanese]Arukoru Kenkyuto Yakubutsu Ison261495919912069537SenguptaASarkarDKRoles of dopamine 2 receptor isoforms and G proteins in ethanol regulated prolactin synthesis and lactotropic cell proliferationPLoS One79e455920122302912310.1371/journal.pone.0045593PMC3445509SoykaMGorigENaberDSerum prolactin increase induced by ethanol—a dose-dependent effect not related to stressPsychoneuroendocrinology1654414461991180529510.1016/0306-4530(91)90009-iValimakiMPelkonenRHarkonenMYlikahriRHormonal changes in noncirrhotic male alcoholics during ethanol withdrawalAlcohol and Alcoholism19323524219846508878

A second component of the stress response is the fight-or-flight response of the sympathetic nervous system, which acts as the first line of defense against stressors. In a stressful situation, a brain region called the amygdala sends out a stress signal to the hypothalamus, which induces the activation of the sympathetic nervous system and the release of the neurotransmitter acetylcholine from preganglionic sympathetic nerves. Acetylcholine, in turn, stimulates the release of the catecholamine hormones epinephrine and norepinephrine from the inner layer (i.e., medulla) of the adrenal gland.^1^ These hormones facilitate an immediate reaction by triggering physiological changes, such as increased heart rate and respiration, and provide the body with a burst of energy through the release of sugar (i.e., glucose) and fat into the bloodstream as energy sources that help the body to respond to the stressors and fight off the threat. This part of the stress response also is regulated by BEP produced from POMC in the hypothalamus, which not only modulates CRH release but also can help decrease the stress response and return the body to a state of homeostasis.^2^ BEP binds with high specificity to different receptors (i.e., μ- and δ-opioid receptors), thereby inhibiting the sympathetic nervous system response to stress. BEP produced from pituitary POMC in response to hypothalamic CRF and AVP, in contrast, circulates in the periphery and has less impact on sympathetic nervous system function (Wynne and Sarkar 2013).

### Alcohol’s Effects on the HPA Axis

Considerable lines of evidence indicate that alcohol consumption affects the stress-response pathways and the HPA axis. Acute exposure to alcohol activates the HPA axis, leading to a dose-related increase in circulating ACTH and glucocorticoids and inducing anxiolytic-like responses (Richardson et al. 2008; Varlinskaya and Spear 2006). Jenkins and Connolly (1968) showed that plasma cortisol levels significantly increased in healthy subjects at alcohol doses exceeding 100 mg/dL. Similarly, healthy men who were in the top percentile of self-reported alcohol consumption had higher levels of excreted cortisol in urine (Thayer et al. 2006). In addition, these researchers reported that the inhibitory control of the HPA axis was impaired in heavy drinkers. Finally, people with a family history of alcohol use disorder (AUD) exhibited hyperresponsiveness of the stress response mediated by the HPA axis (Uhart et al. 2006; Zimmermann et al. 2004).

Similar findings were obtained in animal studies, where acute ethanol administration to rats increased plasma ACTH and corticosterone levels by enhancing CRF release from the hypothalamus (Rasmussen et al. 2000; Rivier and Lee 1996). Neutralization of circulating CRF using specific antibodies inhibited ethanol’s stimulatory actions on ACTH and corticosterone secretion (Rivier and Lee 1996). Additional studies of chronic alcohol administration found an association between HPA axis response and level of alcohol consumption (Richardson et al. 2008). In these analyses, the HPA response after several weeks of daily 30-minute self-administration of alcohol was highest in the animals with the lowest level of consumption (<0.2 mg/kg/session) and most blunted in animals with the highest level of consumption (~1.0 mg/kg/session). Furthermore, chronic alcohol exposure was associated with anxiety-producing–like (i.e., anxiogenic-like) behaviors (King et al. 2006). These studies clearly indicate that chronic exposure to alcohol attenuates basal ACTH and corticosterone levels and increases anxiogenic-like behaviors.

Various mechanisms have been proposed for the blunted HPA axis responsiveness to chronic alcohol consumption. Several of these focus on the relationship between alcohol and CRF expression:

- Alcohol dependence has been shown to be associated with a decrease in CRF mRNA expression (Richardson et al. 2008) as well as reduced responsiveness of the pituitary to CRF (Sarnyai et al. 2001).
- Animal studies using mice that produced no CRF (i.e., CRF knockout mice) found that when the animals were exposed to ethanol (in a continuous- or a limited-access paradigm), they consumed twice as much ethanol as their counterparts with a functional CRF gene. In addition, the knockout mice exhibited a reduced sensitivity to the locomotor-stimulant and rewarding effects of ethanol (Olive et al. 2003).
- Mice lacking a functional CRF1 receptor progressively increased their ethanol intake when subjected to repeated stress; this effect seemed to persist throughout their life (Sillaber et al. 2002).

Numerous studies have suggested that genetically determined differences in the HPA axis stress response, glucocorticoid signaling, and the BEP and opioid system also may be involved in the predisposition for, as well as development and progression of, AUD. However, a discussion of this evidence and the proposed mechanisms is beyond the scope of this article.

### The HPA Axis, Alcohol, and the Immune System

AUDs often are associated with chronic systemic inflammation and high levels of circulating proinflammatory cytokines. Alcohol may induce inflammation through both direct and indirect mechanisms. For example, alcohol metabolism results in the production of reactive oxygen species (ROS) and cell damage that can trigger the production of proinflammatory cytokines (Haorah et al. 2008). Alcohol also may damage the bacterial flora in the gut as well as the intestinal walls, leading to the release and transfer into the blood of bacterial lipopolysaccharides, which play a key role in alcohol-mediated inflammation (Purohit et al. 2008; Wang et al. 2010). A bidirectional interaction between the HPA axis and the immune system also may contribute to alcohol-induced inflammatory reactions. Thus, by binding to their receptors, glucocorticoids can interfere with certain signaling pathways that repress transcription of many inflammatory proteins (Barnes 2006).

In addition, CRF and ACTH have immuno-potentiating and proinflammatory properties (figure 1) (Besedovsky and del Rey 1996). Conversely, interleukins (ILs) and cytokines produced by activated immune cells (i.e., macrophages) can act on the HPA axis and induce CRF and ACTH secretion in an adaptive feedback mechanism (Bateman et al. 1989; Blalock and Costa 1989). This bidirectional interaction between the HPA axis and immune function is essential for survival and for maintaining the body’s homeostasis. However, excessive alcohol exposure compromises HPA axis and immune functions by altering cytokine levels in a variety of tissues, including the brain, with the specific effect on cytokine production depending on the length of exposure. For example, acute exposure to ethanol is associated with suppressed production of certain cytokines (e.g., tumor necrosis factor alpha [TNFα] and IL-1β) (Pruett et al. 2004), whereas chronic exposure induces an increase in the production of proinflammatory cytokines, such as TNFα (Mandrekar et al. 2009; Nagy 2004). The increase in innate immune signaling molecules in the brain associated with chronic alcohol consumption can affect cognitive function and promote alcohol use behaviors.

It has been speculated that dysregulations of HPA axis function caused by chronic alcohol exposure mediates these effects on the immune system (figure 1). Several studies clearly have demonstrated that ethanol exposure during the developmental period induced neurotoxicity and permanent impairments in the HPA axis that were associated with immune dysfunction (Hellemans et al. 2010; Kuhn and Sarkar 2008; Sarkar et al. 2007). Macrophages residing in the brain (i.e., microglia) play an important role in these neurotoxic effects of alcohol (Boyadjieva and Sarkar 2010; Fernandez-Lizarbe et al. 2009).

## Alcohol and the HPG Axis

### Normal Functioning of the HPG Axis

Reproductive function is regulated by a cascade of events that are under the control of the HPG axis. The hypothalamus produces and secretes LHRH, also called gonadotropin-releasing hormone, into the hypothalamic–pituitary portal network. At the anterior pituitary, LHRH stimulates the production and secretion of FSH and LH from gonadotropic cells into the general circulation. These gonadotropins regulate the development of follicles (i.e., folliculogenesis) in females and of sperm (i.e., spermatogenesis) in males. Moreover, each month during the follicular phase of the menstrual cycle, FSH stimulates the development of a dominant follicle in the ovary, which then produces and secretes the hormone estradiol. The rise in estradiol through a feedback mechanism is responsible for the surge in LH and FSH levels that occurs in the middle of the menstrual cycle. LH then induces ovulation and the development of the corpus luteum, which in turn produces and secretes progesterone, an important hormone that helps maintain pregnancy. In the testes, in contrast, LH stimulates testosterone production and release, whereas FSH controls spermatogenesis. HPG axis function is controlled through feedback mechanisms, where testosterone, estrogen, and progesterone control their own production by acting on the hypothalamus and anterior pituitary to inhibit or stimulate the release of LHRH, LH, and FSH (Sarkar 1983).

### Alcohol’s Effects on the HPG Axis

**Figure 2:**
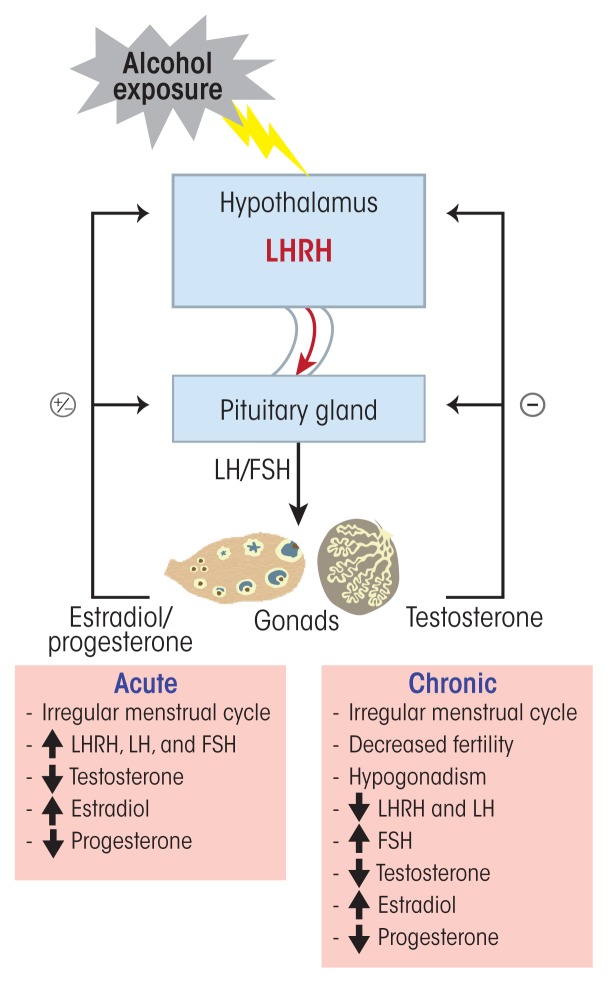
Alcohol’s effects on the hypothalamic–pituitary–gonadal (HPG) axis. Neurons in the hypothalamus release luteinizing hormone–releasing hormone (LHRH) to the hypophyseal-portal blood system. LHRH then stimulates the secretion of gonadotropins (i.e., LH and FSH). During the ovary’s follicular phase, FSH stimulates the development of a dominant follicle, which produces and secretes estradiol. Estradiol then stimulates an LH and FSH surge during midcycle of the menstrual cycle. LH stimulates ovulation and the development of the corpus luteum, which then produces and secretes progesterone. In the testis, LH stimulates testosterone production and release, while FSH controls spermatogenesis. HPG axis function is controlled through feedback loop mechanisms. Testosterone inhibits LHRH, LH, and FSH secretion through negative feedback, whereas estradiol and progesterone both can have negative- and positive-feedback actions, depending on the stage of the ovarian cycle, and can inhibit or stimulate the release of LHRH, LH, and FSH. Acute alcohol exposure results in increased LHRH, LH, FSH, and estradiol and decreased testosterone and progesterone. Chronic alcohol exposure, in contrast, induces a decrease in LHRH, LH, testosterone, and progesterone and an increase in estradiol and FSH. These alcohol-induced hormonal dysregulations cause a multitude of reproductive disorders, such as menstrual cycle irregularity, decreased fertility, and hypogonadism.

Numerous studies have documented alcohol’s diverse deleterious effects on the HPG axis and its hormones (figure 2). The resulting HPG dysfunction observed in people with AUD can be associated with diverse outcomes, including a decreased libido, infertility, and gonadal atrophy. It also is important to note that these deleterious effects are not limited to adult drinkers but may also affect adolescents in puberty who begin to consume alcohol. For more information, see the sidebar “Alcohol’s Effects on the Hypothalamic–Pituitary– Gonadal Axis During Puberty.”

In women, alcohol use can cause a multitude of reproductive disorders, such as irregular menstrual cycles, absence of ovulation (i.e., anovulation), increased risk of spontaneous abortions, and early menopause. Alcohol intake, even as little as five drinks per week, was associated with decreased fecundability in healthy women ages 20–35 (Jensen et al. 1998). Other studies (Mendelson et al. 1988) found that 50 percent of social (i.e., about 3.84 drinks per day) and 60 percent of heavy (i.e., about 7.81 drinks per day) healthy, nondependent drinkers exhibited significant disturbances of their reproductive hormones and menstrual cycle compared with occasional drinkers (i.e., about 1.22 drinks per day). In addition, social drinkers had anovulatory cycles, and 3 of 5 heavy drinkers exhibited excessive levels of prolactin in the blood (i.e., hyperprolactinemia) (Mendelson et al. 1988). Studies have shown that alcohol intake consistently induces an increase in estradiol levels in humans (Mendelson and Mello 1988; Muti et al. 1998) and rodents (Emanuele et al. 2001*a*), possibly as a result of decreased steroid catabolism (Sarkola et al. 1999). These increased estradiol levels could in part explain alcohol’s negative effects on menstrual cycle regularity. Moreover, chronic alcohol has inhibitory actions on LHRH-producing neurons. Thus, exposure to 100 mM ethanol directly inhibited LHRH release from incubated medial basal hypothalamic sections, and this effect was reversed by naltrexone (Lomniczi et al. 2000). These results suggest that alcohol’s effect on LHRH release involves the stimulation of BEP-releasing neurons, which prevent LHRH release by inhibiting nitric oxide synthase. Other studies have shown that long-term moderate alcohol consumption can decrease the number and quality of a woman’s oocytes (i.e., ovarian reserve), which was associated with increased FSH levels (Li et al. 2013).

Extensive research in animals and humans also has documented the deleterious effects of alcohol on male reproductive function, including reduced testosterone levels (figure 2). Acute alcohol intake decreased the circulating levels of LH and testosterone as a result of diminished release of hypothalamic LHRH (Cicero et al. 1982; Dees et al. 1983; Rowe et al. 1974). In contrast, chronic alcohol consumption significantly increased FSH, LH, and estrogen levels but decreased testosterone and progesterone levels in men with AUD compared with men without AUD (Muthusami and Chinnaswamy 2005). The AUD group also had significantly lower semen volume, sperm count, motility, and number of morphologically normal sperm (Muthusami and Chinnaswamy 2005). Several mechanisms may contribute to alcohol’s effects on the various hormones involved in the male HPG axis:

- The activity of the enzyme aromatase, which converts androgens to estrogens, especially in the liver, is increased by ethanol (Purohit 2000). This mechanism may explain why alcohol abuse results in hypogonadism even in the absence of liver disease.
- In men with AUD and cirrhosis, a decrease in IGF-1 bioavailability as a result of liver disease contributes at least in part to the elevated circulating levels of estradiol and estrone (Martinez-Riera et al. 1995) and the development of hypogonadism (Castilla-Cortazar et al. 2000) since IGF-1 can stimulate testosterone synthesis and spermatogenesis (Roser 2008).
- ROS produced during alcohol metabolism may cause cell damage in the testes (Emanuele et al. 2001*b*). The testicular alcohol-inducible cytochrome P450 2E1, which is involved in the generation of ROS as well as hydroxyl ethyl free radicals, was shown to be elevated in testes of rats chronically exposed to ethanol (Shayakhmetova et al. 2013).
- The alcohol metabolite acetaldehyde can disrupt testosterone production by inhibiting protein kinase C, a key enzyme in testosterone synthesis (Chiao and Van Thiel 1983).
- Nitric oxide, which is synthesized in the testes by nitric oxide synthase, is another proposed player in the alcohol-induced reduction of testosterone production. Inhibition of nitric oxide synthase prevents the alcohol-induced decrease in testosterone (Adams et al. 1992).

## Alcohol and the HPT Axis

### Normal Functioning of the HPT Axis

The HPT axis is responsible for maintaining normal circulating levels of the thyroid hormones thyroxin (T4) and its active form, triiodothyronine (T3). These two hormones affect every cell and organ in the body, primarily regulating different metabolic processes that influence how cells use different energetic compounds (i.e., proteins, fats, and carbohydrates). When circulating levels of thyroid hormones are low, the hypothalamus responds by releasing TRH, which then stimulates thyrotropic cells in the anterior pituitary to produce and secrete TSH. This hormone, in turn, promotes the synthesis and secretion of T4 and T3 from the follicular cells of the thyroid gland. Iodine is essential to T4 and T3 production, with T4 containing four, and T3 containing three, iodine atoms. Although both T4 and T3 are secreted by the thyroid following TSH stimulation, 80 percent of circulating T3 is derived from the conversion of T4 by enzymes called deiodinases in the liver. Like the HPA and HPG axes, the HPT axis is regulated by negative-feedback loops where T4 and T3 act back on the hypothalamus and the pituitary to control their own release by inhibiting TRH and TSH secretion.

### Alcohol’s Effects on the HPT Axis

**Figure 3:**
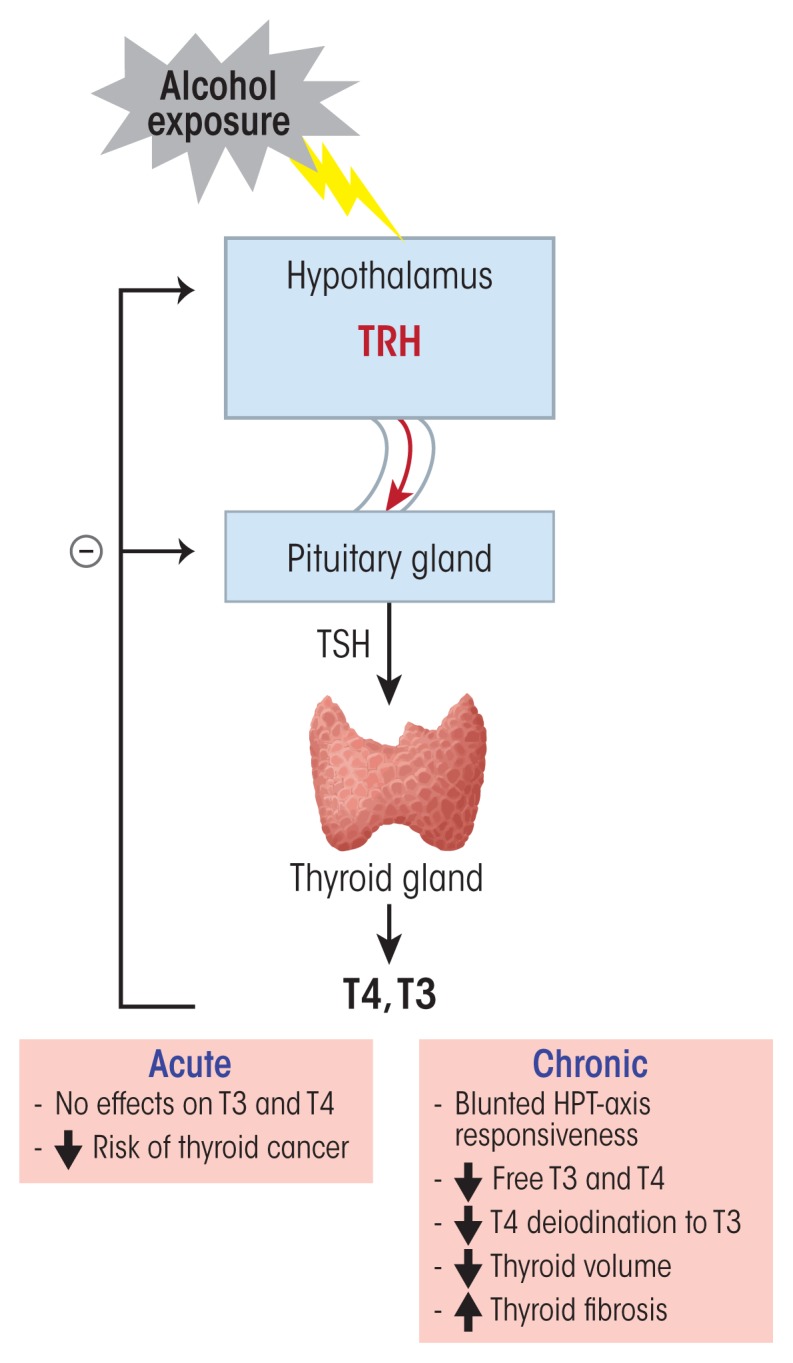
Alcohol’s effects on the hypothalamic–pituitary–thyroid (HPT) axis. Thyrotropin-releasing hormone (TRH) released from neurons in the hypothalamus stimulates thyrotropic cells in the anterior pituitary to produce and secrete thyroid-stimulating hormone (TSH). TSH then stimulates the synthesis and secretion of thyroxin (T4) and its active form, triiodothyronine (T3), from the follicular cells of the thyroid gland. Circulating T3 comes from conversion of T4 by enzymes called deiodinases in the liver. T3 and T4 can control their own release by negative feedback at the hypothalamus and the pituitary and inhibit TRH and TSH release. Acute alcohol exposure has no effect on HPT-axis function. However, chronic alcohol exposure leads to a blunted TSH response to TRH, as well as to decreased free T3 and T4, decreased deiodination of T4 to T3, decreased thyroid volume, and increased thyroid fibrosis.

Numerous studies have described HPT axis dysfunction in people with AUD (see figure 3). For example, these individuals consistently exhibit a reduced or absent response of TSH to TRH (Sellman and Joyce 1992). A blunted TSH response also was observed during early withdrawal and was positively correlated with severity of withdrawal symptoms; in fact, it may be an important predictor of relapse (Pienaar et al. 1995). However, conflicting changes in peripheral thyroid hormones in response to alcohol exposure and withdrawal have been reported. T4 and T3 circulate in two forms, a protein-bound inactive form and a free, readily available active form. Some studies found normal concentrations of total plasma T4 (tT4) during early withdrawal (Majumdar et al. 1981), whereas others found significantly reduced tT4 levels (Valimaki et al. 1984). The levels of free T4 and T3, however, were lower in people with AUD during withdrawal and early abstinence compared with nonalcoholic healthy control subjects (Hegedus et al. 1988). Additional analyses identified a significant positive correlation between free T3 and alcohol-seeking behaviors in alcohol-dependent individuals (Aoun et al. 2015), supporting the hypothesis of a relationship between alcohol dependence and thyroid dysfunction. This thyroid dysfunction can recover after longer periods of abstinence, with thyroid hormones and the TSH response to TRH returning to normal levels (Pienaar et al. 1995). Moreover, people who relapsed and returned to their alcohol-drinking behavior again exhibited lower T4 and T3 levels and a blunted TSH response to TRH (Heinz et al. 1996). Animal studies have yielded similar results. Chronic exposure of adult male rats to ethanol (10 percent weight/volume) for 40 days induced a significant decrease in total T4 and T3, free T4 and T3, as well as basal TSH levels (Mason et al. 1988).

**Alcohol’s Effects on the Hypothalamic–Pituitary–Gonadal Axis During Puberty**

Little research has assessed the effects of alcohol use on the hypothalamic–pituitary–gonadal (HPG) axis during puberty in humans. Initiation and progression of puberty are controlled by signals from the central nervous system that stimulate the pulsatile diurnal secretion of luteinizing hormone-releasing hormone (LHRH) from the hypothalamus into the hypothalamic–pituitary portal system (Sarkar and Fink 1979; Sarkar et al. 1976). LHRH then triggers the pituitary to secrete luteinizing hormone (LH) and follicle-stimulating hormone (FSH), resulting in subsequent ovarian maturation (Plant 2015). During childhood, the LHRH surge is repressed through inhibitory signals in the hypothalamus mediated by γ-aminobutyric acid and opioid peptides (Terasawa and Fernandez 2001). During puberty, however, LHRH release is triggered by a variety of stimulatory agents, such as insulin-like growth factor-1 (IGF-1) (Hiney and Dees 1991), norepinephrine (Sarkar et al. 1981), leptin (Dearth et al. 2000), transforming growth factor alpha (Ojeda et al. 1990), and kisspeptins (Navarro et al. 2005).

Human studies have documented that moderate alcohol consumption induces disruptions in normal hormone levels during puberty, including a decrease in estrogen levels in adolescent girls that was sustained for long periods of time (Block et al. 1993). Similar, alcohol abuse induced a significant reduction in testosterone, LH, and FSH levels in adolescent boys (Diamond et al. 1986). Animal studies on rodents and monkeys have helped to understand and identify the mechanisms involved in these alcohol-mediated disruptions of puberty-related processes. Bo and colleagues (1982) reported that alcohol administration to prepubertal female rats induced a marked delay in vaginal opening. This delay could be prevented by naltrexone, an antagonist of the opioid receptors (Emanuele et al. 2002), suggesting that alcohol’s effects during puberty partly may result from an increased opioid restraint on the normal progression of pubertal processes. Another proposed mechanism for the alcohol-induced decrease in LH secretion during puberty is that even though the hypothalamus produced more LHRH, the release of the hormone to the pituitary gland was diminished (Dees and Skelley 1990). This effect may result, at least in part, from altered release of prostaglandin E2 (Hiney and Dees 1991), which normally mediates stimulation of LHRH release by norepinephrine. In addition, alcohol exposure induces an increase in hypothalamic growth hormone (GH)-releasing hormone content that also is associated with diminished release of the hormone and, therefore, reduced ability to stimulate GH secretion from the anterior pituitary (Dees and Skelley 1990). These effects of alcohol exposure on GH were associated with a decrease in circulating IGF-1, which could explain the growth impairments observed in animals exposed to alcohol (Srivastava et al. 1995). In studies in rhesus macaques, administration of alcohol (2 g/kg) for 12 months to immature females resulted in suppression of the nightly increase in circulating GH that occurs during late juvenile development (Dees et al. 2000). This effect was associated with a significant decline in circulating IGF-1, LH, and estrogen and was most pronounced at 32 months of age. The reduced hormone levels affected the monthly pattern of menstruation in the rhesus macaques and induced a lengthening of the intervals between menses in the alcohol-exposed monkeys (Dees et al. 2000).

Taken together, these findings clearly show that the activities of the HPG and GH/IGF-1 axes during puberty are closely interconnected. This is further demonstrated by observations that estrogen can stimulate GH secretion (Mauras et al. 1996) and that IGF-1 can stimulate LHRH secretion (Hiney and Dees 1991), suggesting that activation of the HPG axis leads to both sexual maturation and a growth spurt mediated through estrogen-induced stimulation of the GH/IGF-1 axis. Therefore, alcohol-induced disturbances in the activity of the HPG axis during this critical stage of human development could have far-reaching consequences on reproductive function as well as growth that might persist through adult life.

ReferencesBlockGDYamamotoMEMallickAStycheAEffects on pubertal hormones by ethanol abuse in adolescentsAlcoholism: Clinical and Experimental Research175051993BoWJKruegerWARudeenPKSymmesSKEthanol-induced alterations in the morphology and function of the rat ovaryAnatomical Record20222552601982719983410.1002/ar.1092020210DearthRKHineyJKDeesWLLeptin acts centrally to induce the prepubertal secretion of luteinizing hormone in the female ratPeptides21338739220001079322110.1016/s0196-9781(00)00157-1DeesWLSkelleyCWEffects of ethanol during the onset of female pubertyNeuroendocrinology51164691990210608910.1159/000125317DeesWLDissenGAHineyJKAlcohol ingestion inhibits the increased secretion of puberty-related hormones in the developing female rhesus monkeyEndocrinology14141325133120001074663510.1210/endo.141.4.7413DiamondFJrRingenbergLMacDonaldDEffects of drug and alcohol abuse upon pituitary-testicular function in adolescent malesJournal of Adolescent Health Care7128331986293551510.1016/s0197-0070(86)80091-2EmanueleNRenJLaPagliaNEtOH disrupts female mammalian puberty: Age and opiate dependenceEndocrine18324725420021245031610.1385/ENDO:18:3:247HineyJKDeesWLEthanol inhibits luteinizing hormone-releasing hormone release from the median eminence of prepubertal female rats in vitro: Investigation of its actions on norepinephrine and prostaglandin-E2Endocrinology1283140414081991199916210.1210/endo-128-3-1404MaurasNRogolADHaymondMWVeldhuisJDSex steroids, growth hormone, insulin-like growth factor-1: Neuroendocrine and metabolic regulation in pubertyHormone Research451–274801996874212310.1159/000184763NavarroVMCastellanoJMFernandez-FernandezRCharacterization of the potent luteinizing hormone-releasing activity of KiSS-1 peptide, the natural ligand of GPR54Endocrinology146115616320051537502810.1210/en.2004-0836OjedaSRUrbanskiHFCostaMEInvolvement of transforming growth factor alpha in the release of luteinizing hormone-releasing hormone from the developing female hypothalamusProceedings of the National Academy of Sciences of the United States of America8724969897021990226362110.1073/pnas.87.24.9698PMC55240PlantTMNeuroendocrine control of the onset of pubertyFrontiers in Neuroendocrinology38738820152591322010.1016/j.yfrne.2015.04.002PMC4457677SarkarDKFinkGMechanism of the first spontaneous gonadotrophin surge and that induced by pregnant mare serum and effects of neonatal androgen in ratsJournal of Endocrinology833339354197939526710.1677/joe.0.0830339SarkarDKChiappaSAFinkGSherwoodNMGonadotropin-releasing hormone surge in pro-oestrous ratsNature2645585461463197679473710.1038/264461a0SrivastavaVHineyJKNybergCLDeesWLEffect of ethanol on the synthesis of insulin-like growth factor 1 (IGF-1) and the IGF-1 receptor in late prepubertal female rats: A correlation with serum IGF-1Alcoholism: Clinical and Experimental Research196146714731995874981210.1111/j.1530-0277.1995.tb01009.xTerasawaEFernandezDLNeurobiological mechanisms of the onset of puberty in primatesEndocrine Reviews22111115120011115981810.1210/edrv.22.1.0418

Several mechanisms have been proposed to explain the blunted TSH response to TRH in people with AUD. For example, several studies suggest that the number of TRH receptors in the pituitary is reduced as a result of increased TRH secretion (Aoun et al. 2015; Herman 2002). A role for increased TRH section in blunting the TSH response also is supported by observations that abstinent patients with AUD who had a severely blunted TSH response to TRH showed increased levels of TRH in the cerebrospinal fluid (Adinoff et al. 1991). In rats, chronic alcohol exposure induced an increase in TRH mRNA in neurons of the PVN, but the animals no longer responded to peripheral stimulation of thyroid hormone secretion by exposure to cold (Zoeller et al. 1996). This suggests that chronic exposure to ethanol induces dysfunction of the thyroid gland, which then is no longer able to properly respond to TRH stimulation.

Direct actions of ethanol on thyroid hormone metabolism, specifically on the activity of enzymes that catalyze the conversion of T4 to T3 (i.e., 5′II deiodinase) or inactivate T3 to 3,3′-T2 (i.e., 5-II deiodinase), also have been proposed. In a study comparing “behaviorally dependent” and ethanol-exposed but “nondependent” rats, Baumgartner and colleagues (1997) found that the activity of 5′II deiodinase was elevated in the frontal cortex in both groups of rats. The activity of 5-II deiodinase, however, was only inhibited in the amygdala of the rats that were behaviorally dependent on ethanol but was normal in the non-dependent rats. As a result, intracellular T3 levels were increased, and this increase of intracellular T3 in the amygdala might be involved in the development of dependence behaviors to alcohol (Baumgartner et al. 1997). The role of changes in thyroid hormone levels in the development of AUD also is supported by findings that a functionally significant genetic variant (i.e., single nucleotide polymorphism) in the deiodinase type II (D2) gene was associated with drinking behavior in alcohol-dependent individuals (Lee et al. 2015).

Chronic alcohol use also had a direct toxic effect on the thyroid gland, inducing a dose-dependent significant reduction in thyroid volume and increase in thyroid fibrosis in alcohol-dependent individuals (Hegedus et al. 1988). These effects were associated with reductions in total and free T3 levels, although the concentrations of total and free T4 as well as of TSH remained unchanged (Hegedus et al. 1988). In contrast to these effects of chronic alcohol use on thyroid hormones, moderate alcohol consumption was shown to reduce the risk of developing thyroid cancer. Several studies, including the large NIH–AARP Diet and Health Study that followed 490,000 participants (males and females) over 7.5 years, have shown a significant reduction in the risk of developing all types of thyroid cancers in people who consumed two or more alcoholic drinks per day, especially in men. However, the effects differed between different subtypes of thyroid cancer, with a stronger inverse association for papillary thyroid cancer (relative risk = 0.58) compared with follicular thyroid cancer (relative risk = 0.86) (Meinhold et al. 2009). Furthermore, in a study of 4,649 healthy individuals who were exposed to increasing levels of alcohol, Knudsen and colleagues (2001) found an association between a reduced thyroid gland volume and a lower risk of developing goiter or solitary nodules.

## Alcohol and the GH/IGF-1 Axis

### Normal Functioning of the GH/IGF-1 Axis

Like the other hormone systems discussed so far, the GH/IGF-1 axis is under the control of the hypothalamus. Growth hormone–releasing hormone (GHRH) secreted from cells in the arcuate and ventromedial nuclei of the hypothalamus into the hypophyseal portal system acts on somatotropic cells in the anterior pituitary, stimulating them to synthesize and release GH into the general circulation. GH is essential to the growth of all tissues in the body. It stimulates protein synthesis and increases fat metabolism to provide the necessary energy for growth. GH binds to specific receptors on target tissues and directly affects cell function or it stimulates IGF-1 production and secretion, especially from the liver, the principal production site for this factor. IGF-1 then is either released into the general circulation, where it is bound to large circulatory binding proteins that regulate its delivery to target tissues, or it mediates the anabolic effects of GH through paracrine and autocrine mechanisms. At birth, plasma IGF-1 levels are at 50 percent of the adult levels and gradually increase throughout childhood with a spike during puberty, when IGF-1 plays a critical role in reproductive-organ maturation and long-bone growth. After puberty, the levels again decrease slowly to reach the adult level. IGF-1 can control its own secretion through negative feedback at the level of the hypothalamus and pituitary by reducing GH synthesis and release.

Another hormone called somatostatin, which is secreted from the PVN of the hypothalamus, also acts on the pituitary and inhibits GH secretion. Thus, the amount of GH secreted by the anterior pituitary is tightly regulated by GHRH, IGF-1, and somatostatin. Together, GH and IGF-1 regulate important physiological processes in the body, such as pre- and postnatal growth and development (Giustina et al. 2008) and carbohydrate and lipid metabolism (Moller and Jorgensen 2009).

### Alcohol’s Effects on the GH/IGF-1 Axis

**Figure 4:**
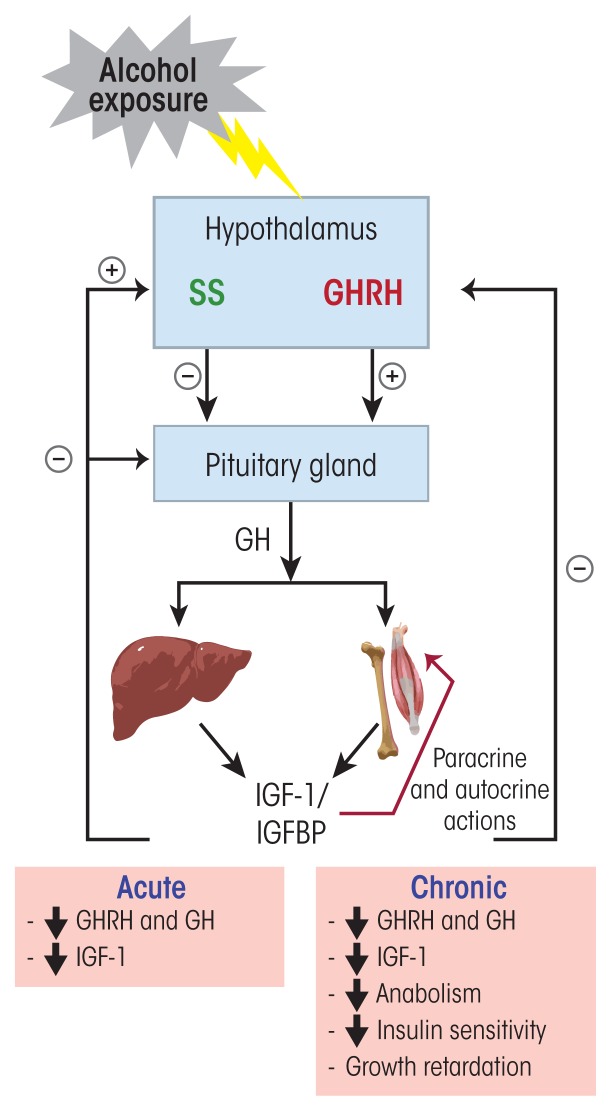
Alcohol’s effects on the growth hormone–insulin-like growth factor-1 (GH/IGF-1) axis. Growth hormone (GH)-releasing hormone (GHRH) secreted from neurons in the hypothalamus acts on somatotropic cells in the anterior pituitary and stimulates the production and release of GH into the circulation. GH can act on target tissues and directly affect their function or it can stimulate IGF-1 production and secretion from these target tissues, especially from the liver. IGF-1 then is either released into the general circulation, where it circulates bound to IGF binding proteins (IGFBP), or it can mediate GH anabolic effects on target tissues through paracrine and autocrine actions. Through negative feedback at the hypothalamus and pituitary, IGF-1 can reduce GHRH and GH secretion. Somatostatin (SS), secreted in the paraventricular nucleus of the hypothalamus, also acts on the pituitary and inhibits GH secretion. IGF-1 stimulates SS secretion. Acute and chronic alcohol exposure leads to decreased GHRH, GH, and IGF-1 secretion.

Numerous studies in both humans and experimental animals have shown that acute and chronic alcohol exposure has a variety of effects on the GH/IGF-1 axis (figure 4). For example, alcohol exposure reduces circulating GH and IGF-1 levels. Acute exposure of healthy men to ethanol (1.5 g/kg) reduced the nightly peak of GH secretion (Valimaki et al. 1987). This effect did not seem to be mediated through a direct action of ethanol on the pituitary that would have rendered it less sensitive to GHRH, because intravenous injection of exogenous GHRH induced an increase in GH secretion in both ethanol-exposed (1 g/kg) and control men (Valimaki et al. 1987). Similarly, De Marinis and colleagues (1993), using an agent that can stimulate GHRH secretion (i.e., clonidine), demonstrated that the pituitary response to GHRH was intact in abstinent alcoholics. Other studies evaluated alcohol’s effects on numerous other factors that regulate GH secretion either through direct actions on the anterior pituitary or by modulating GHRH and somatostatin release from the hypothalamus. The analyses demonstrated that during early abstinence, the GH response to these different secretagogues, which include such neurotransmitters as dopamine, norepinephrine, acetylcholine, γ-aminobutyric acid (GABA), and serotonin, also is altered. For example, men with AUD exhibited impairments both in the serotonin-mediated stimulation of GH secretion (Coiro and Vescovi 1995) and in melatonin’s effect on basal and hypoglycemia-induced GH secretion (Coiro and Vescovi 1998) during early abstinence. Moreover, intravenous injection of 10 mg diazepam, an allosteric modulator of GABA receptor function, had no effect on GH secretion in men with AUD who had maintained a 5-week abstinence, whereas control subjects without AUD showed a striking increase of GH secretion in response to diazepam (Vescovi and Coiro 1999). Finally, alcohol interferes with the normal release pattern of GH. The hormone normally is secreted in a pulsatile manner, with the major secretory episode of GH occurring shortly after sleep onset, during the first period of slow-wave sleep. Studies have identified a consistent and robust relationship between slow-wave sleep and increased GH secretion as well as between sleep disturbances and decreased GH secretion (Van Cauter et al. 2004). Alcohol-dependent individuals have been shown to have lower levels of slow-wave sleep power that was associated with lower levels of GH release compared with normal control subjects (Lands 1999).

**Alcohol and Other Endocrine Tissues**

In addition to the brain areas and organs involved in the main hormone axes in the body that are discussed in this article, several other tissues also produce and secrete hormones that regulate crucial body functions, including the pancreas and fat (i.e., adipose) tissue. Alcohol exposure also can interfere with these hormonal systems.

**The Endocrine Pancreas**

The pancreas, which lies behind the stomach, serves two major functions. First, acinar cells secrete digestive enzymes into the small intestine, thereby supporting digestion. Second, islet cells dispersed throughout the whole pancreas have an endocrine activity by producing hormones (i.e., insulin and glucagon) that regulate blood glucose levels. These islet cells can be further subdivided into α- and β-cells. The α-cells produce glucagon, which raises blood glucose levels by stimulating the liver to metabolize glycogen into glucose molecules and to release the glucose into the blood. In addition, glucagon stimulates the adipose tissue to metabolize triglycerides into glucose, which then is released into the blood. Conversely, the β-cells of the pancreas produce insulin, which lowers blood glucose levels after a meal by stimulating the absorption of glucose by liver, muscle, and adipose tissues and promoting the storage of glucose in the form of glycogen in these tissues. The endocrine function of the pancreas primarily is controlled by both the sympathetic and the parasympathetic divisions of the autonomic nervous system.

***Alcohol’s Effects on the Endocrine Pancreas***

Heavy alcohol drinking can induce the development of inflammation of the pancreas (i.e., pancreatitis), most commonly in acinar cells. However, the inflammatory aspect of this disease also can damage islet cells and, therefore, the endocrine pancreas (Apte et al. 1997). Chronic alcohol consumption also is a risk factor for the development of pancreatic cancer, with moderate to heavy consumption increasing the risk both alone and in combination with other risk factors, such as tobacco and obesity (de Menezes et al. 2013; Haas et al. 2012). One type of pancreatic cancer called ductal adenocarcinoma has a very aggressive behavior with a 5-year survival rate of less than 4 percent (Welsch et al. 2006).

Chronic alcohol consumption also is a known independent risk factor for the development of type 2 diabetes (Hodge et al. 1993; Holbrook et al. 1990; Wei et al. 2000). This syndrome is characterized by impaired glucose metabolism with high blood glucose levels (i.e., hyperglycemia) and peripheral insulin resistance. The relationship between alcohol consumption and the risk of type 2 diabetes is “U” shaped—that is, risk is lower with moderate alcohol consumption than with either abstention or high alcohol consumption. Thus, the risk was reduced by 30 percent in moderate drinkers compared with abstainers, whereas no risk reduction was observed in heavy drinkers consuming 48 grams of ethanol (i.e., 3 to 4 drinks) per day or more (Koppes et al. 2005). Moderate alcohol use may have protective effects by enhancing peripheral insulin sensitivity (Conigrave et al. 2001; Tomie Furuya et al. 2005). Some studies have shown that moderate alcohol consumption improves peripheral insulin sensitivity without affecting insulin secretion from pancreatic β-cells (Avogaro et al. 2004), whereas others determined a reduced basal insulin secretion rate associated with a lower fasting plasma glucagon concentration (Bonnet et al. 2012). The beneficial metabolic effects of moderate alcohol use on insulin sensitivity and glucose homeostasis therefore might explain the significant reduction in the risk of development of type 2 diabetes and of cardiovascular disorders (Avogaro et al. 2004; Bantle et al. 2008).

Heavy alcohol consumption, in contrast, has several detrimental effects resulting in impaired control of blood glucose levels. In addition to its effects on peripheral tissues, such as adipose tissue and the liver, where it induces insulin resistance, heavy drinking also negatively affects pancreatic β-cell function. In a study by Patto and colleagues (1993), chronic drinkers exhibited a decreased insulin-secretion response to glucose compared with the control group. When the investigators measured the total integrated response values for secreted insulin and for C-peptide^1^ following oral or intravenous glucose administration in these two groups, both values were significantly lower in the chronic drinkers compared with the control group. Moreover, in both groups the total integrated response value for insulin was significantly higher after oral glucose administration than after intravenous administration, suggesting a potentiating incretin^2^ effect on insulin secretion. These findings clearly indicate that chronic alcohol exposure induces a β-cell dysfunction and not an enteroinsular incretin dysfunction, because the decrease in insulin response compared with the control group also was observed when glucose was administered intravenously.

Animal studies demonstrated that mice exposed to chronic alcohol for 8 to 10 weeks developed impairments in fasting glucose levels and exhibited an increase in β-cell apoptosis, which were associated with diminished insulin secretion (Kim et al. 2010). The investigators suggested that alcohol exposure led to a downregulation and inactivation of the enzyme glucokinase, which acts as a β-cell sensor for blood glucose levels. Glucokinase is involved in glucose metabolism that leads to increased production of adenosine-triphosphate, a necessary step in insulin secretion by β-cells. The researchers also detected a decrease in the glucose transporter Glut2 in β-cells as well as a decrease in insulin synthesis, further exacerbating the effects of chronic alcohol exposure.

More recently, Wang and colleagues (2014) reported that intraperitoneal administration of ethanol (3g/kg body weight) to mice resulted in an impaired glucose metabolism, which was associated with decreased expression of two subunits (i.e., α1 and δ-subunits) of the type A gamma-aminobutyric acid (GABA) receptors on pancreatic β-cells. This could account at least for part of the alcohol-induced impairment in β-cell function, because activation of GABA receptors in pancreatic β-cells increases insulin secretion (Bansal et al. 2011), has a protective and regenerative effect on β-cells, and decreases cell apoptosis in cultured islet cells (Dong et al. 2006). The investigators further showed that acute treatment of cultured rat β-cells (i.e., the INS-1 cell line) with 60 mM ethanol interfered with GABA-mediated cell activation as well as insulin secretion and that these effects could be prevented by pretreating the cultured cells with GABA (100 mM), further supporting the theory that alcohol’s effects on β-cells and insulin production are mediated at least in part by GABA signaling (Wang et al. 2014). In addition, experiments in another cultured β-cell line indicated that heavy alcohol consumption may induce β-cell dysfunction in type 2 diabetes by increasing the production of reactive oxygen species and inducing apoptosis in the cells (Dembele et al. 2009).

All of these studies clearly show that heavy alcohol consumption has deleterious effects on pancreatic β-cell function and glucose homeostasis. However, more studies are needed to specify the mechanisms by which chronic alcohol affects β-cell function.

**Endocrine Adipose Tissue**

There are two types of adipose tissue—white adipose tissue (WAT) and brown adipose tissue (BAT)—that differ in their morphology and function. For a long time, WAT had been considered a passive reservoir for energy storage. Over the last decade, however, numerous studies have demonstrated that WAT is a dynamically active endocrine organ that can produce and secrete biologically active peptides and proteins called adipokines, which have autocrine, paracrine, and endocrine actions. In fact, WAT may be the largest endocrine organ in mammals and can be found in individual pads in different locations throughout the body, both near other organs (i.e., viscerally) and under the skin (i.e., subcutaneously). Depending on its location, WAT synthesizes and secretes different sets of adipokines (Coelho et al. 2013). Since the discovery of leptin (Zhang et al. 1994), multiple adipokines released by WAT have been identified, including hormones, growth factors, and cytokines (Coelho et al. 2013).

WAT also expresses several receptors that allow it to respond to signals from other hormone systems and from the central nervous system. Through these different communication pathways, WAT can influence the function of many tissues, such as hypothalamus, pancreas, skeletal muscle, and immune system. In addition, WAT can coordinate numerous important biological processes through its various adipokines, such as food intake and body weight (leptin), glucose homeostasis (adiponectin and resistin), lipid metabolism, pro- and anti-inflammatory functions (tumor necrosis factor alpha [TNFα] and interleukin-6 [IL-6]), as well as reproductive functions (Campfield et al. 1996; Coelho et al. 2013).

BAT, on the other hand, is present at birth but is almost absent in adult mammals. Brown adipocytes are smaller than white adipocytes, have numerous mitochondria, and specialize in heat production through oxidation of fatty acids (i.e., thermogenesis). However, recent direct and indirect evidence also suggests a potential endocrine role for BAT (Villarroya et al. 2013). Thus, BAT was shown to release factors such as IGF-1, fibroblast growth factor-2, IL-1α, IL-6, bone morphogenetic protein-8b, and lipocalin prostaglandin D synthase that primarily have autocrine or paracrine actions (Villarroya et al. 2013). The only known endocrine factor released by BAT is the active thyroid hormone T3. Upon thermogenic activation, the type II thyroxine 5′-deiodinase enzyme, which is expressed specifically in BAT, converts T4 into T3 (de Jesus et al. 2001).

***Alcohol’s Effects on Endocrine Adipose Tissue***

Although the results have not been consistent, numerous studies have shown that alcohol consumption can change adipokine levels. For example, studies found that leptin levels were increased (Nicolas et al. 2001; Obradovic and Meadows 2002), decreased (Calissendorff et al. 2004), or remained unchanged (Beulens et al. 2008; Strbak et al. 1998) by alcohol exposure. Another adipokine is adiponectin, which is produced and secreted exclusively by WAT and has antidiabetogenic and anti-inflammatory effects. Its production and actions are regulated by TNFα, with the two compounds suppressing each other’s production and antagonizing each other’s actions in target tissues (Maeda et al. 2002). Moderate alcohol consumption can increase adiponectin plasma levels, which is associated with a significant increase in insulin sensitivity (Sierksma et al. 2004; Thamer et al. 2004); the extent of this effect, however, depends on the frequency of alcohol administration. In a study comparing the effects of exposure of high-fat–fed rats to 5 g/kg body weight ethanol per day delivered either by twice-daily administration via a gastric tube or through free-access drinking, Feng and colleagues (2012) demonstrated greater improvement of insulin sensitivity with twice-daily ethanol administration. Accordingly, adiponectin plasma levels were significantly increased in the twice-daily administration group compared with the free-access group. The researchers suggested that ethanol concentrations in the blood might be an important factor influencing adiponectin secretion and, consequently, insulin sensitivity.

**Figure:**
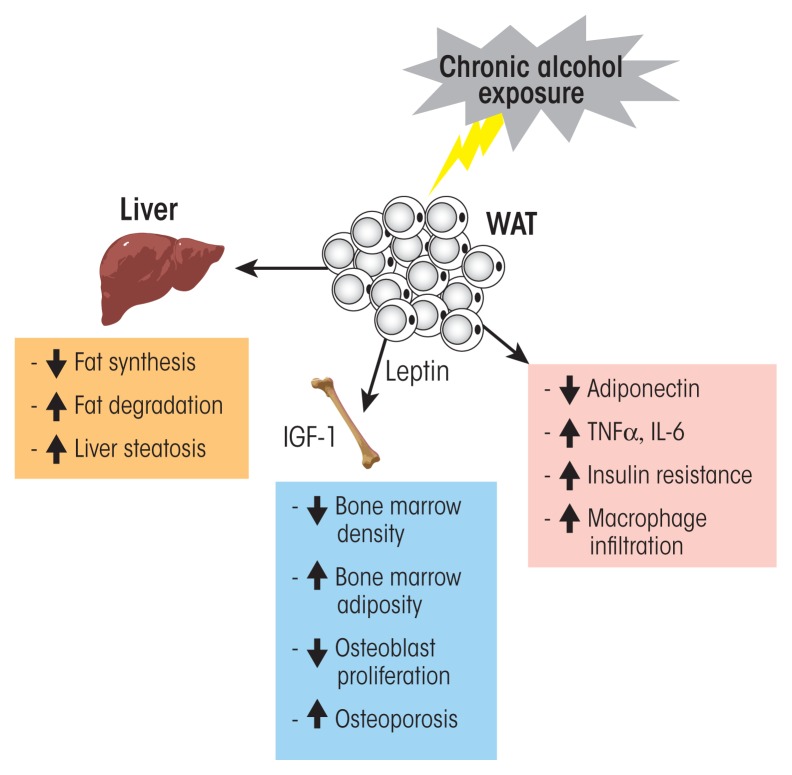
Alcohol and the endocrine white adipose tissue (WAT). WAT is a dynamically active endocrine organ that produces and secretes adipokines, including hormones, growth factors, and cytokines. These factors, through autocrine, paracrine, and endocrine actions, can influence the function of many tissues and coordinate numerous important biological processes such as food intake, glucose homeostasis, lipid metabolism, and pro- and anti-inflammatory functions. Acute and moderate alcohol exposure induces an increase in circulating adiponectin levels, which is associated with decreased insulin resistance. Chronic alcohol exposure induces a decrease in adiponectin, an increase in macrophage infiltration and proinflammatory cytokine secretion (e.g., tumor necrosis factor alpha (TNFα) and interleukin-6 [IL-6]) and insulin resistance. Chronic alcohol exposure also increases the risk of fatty liver (i.e., steatosis).

One proposed mechanism for the adiponectin-mediated improvement in insulin sensitivity is that the increase in adiponectin causes a decrease in plasma levels of TNFα (Ouchi et al. 2000; Yokota et al. 2000). Conversely, decreasing adiponectin levels would be expected to result in increasing TNFα levels. High circulating TNFα levels, in turn, have been implicated in the development of peripheral insulin resistance (Hotamisligil et al. 1995). Chronic alcohol consumption can significantly decrease adiponectin levels (Xu et al. 2003).^3^ Thus, male rats that had received ethanol for 4 weeks exhibited significantly decreased mRNA levels of adiponectin and retinol binding protein 4 but increased mRNA levels of monocyte chemoattractant protein 1, TNFα, and IL-6 in epididymal adipose tissue. These changes were associated with increased macrophage infiltration into adipose tissue and the development of insulin resistance (see figure) (Kang et al. 2007).

In addition, studies have suggested that reduced adiponectin expression could play an important role in the development of alcohol-induced liver damage (Xu et al. 2003). Alcoholic fatty liver (i.e., steatosis) is one of the most prevalent forms of chronic liver diseases caused by alcohol abuse; it is characterized by the excessive accumulation of fat in the liver and can progress to more severe forms of liver injury, such as steatohepatitis, fibrosis, and cirrhosis. Adiponectin’s protective effects on the liver are believed to be mediated through its actions on hepatic signaling molecules involved in enhanced fat oxidation and reduced lipid synthesis (Rogers et al. 2008; Xu et al. 2003). A recent study assessed the serum concentrations of total adiponectin, leptin, and resistin in male and female patients with chronic alcohol abuse and different degrees of liver dysfunction (Kasztelan-Szczerbinska et al. 2013). The analyses found elevated total levels of adiponectin and resistin in patients with alcoholic liver disease (ALD) compared with control subjects. Also, women with ALD had lower leptin levels than did control subjects, whereas there were no significant differences in leptin concentrations in males with and without ALD. Gender-related differences in serum leptin concentrations may influence the clinical course of ALD, which differs in males and females. It is possible that metabolic alterations caused by ethanol in the course of ALD, by differentially modulating leptin secretion, may be responsible for different clinical presentations of the disease in females and males (Kasztelan-Szczerbinska et al. 2013). However, more studies are needed to help with our understanding of the adipose tissue pathology associated with alcohol abuse.

1C-peptide is a chain of 31 amino acids that during insulin synthesis connects the two parts, or chains, of the insulin molecule in a precursor molecule. During final processing of the insulin molecule, the C-peptide is removed to yield the functional insulin molecule with its two chains.2Incretin is a hormone secreted by the wall of the intestine that acts on the pancreas to regulate insulin production after glucose administration. This so-called enteroinsular signaling pathway can therefore only occur after oral glucose administration, which results in increased glucose levels in the intestine, but not after intravenous administration, which bypasses the intestine.3The increased TNFα levels associated with decreased adiponectin also may play a role in the development of liver disease. TNFα production was increased in adipose tissue at early stages of alcoholic fatty liver, resulting in increases in both circulating and local TNFα levels (Lin et al. 1998).

ReferencesApteMVWilsonJSKorstenMAAlcohol-related pancreatic damage: Mechanisms and treatmentAlcohol Health & Research World2111320199715706759PMC6826792AvogaroAWatanabeRMDall’ArcheAAcute alcohol consumption improves insulin action without affecting insulin secretion in type 2 diabetic subjectsDiabetes Care2761369137420041516179010.2337/diacare.27.6.1369BansalPWangSLiuSGABA coordinates with insulin in regulating secretory function in pancreatic INS-1 beta-cellsPLoS One610e2622520112203182510.1371/journal.pone.0026225PMC3198728BantleAEThomasWBantleJPMetabolic effects of alcohol in the form of wine in persons with type 2 diabetes mellitusMetabolism57224124520081819105510.1016/j.metabol.2007.09.007PMC2238804BeulensJWde ZoeteECKokFJEffect of moderate alcohol consumption on adipokines and insulin sensitivity in lean and overweight men: A diet intervention studyEuropean Journal of Clinical Nutrition6291098110520081755424610.1038/sj.ejcn.1602821BonnetFDisseELavilleMModerate alcohol consumption is associated with improved insulin sensitivity, reduced basal insulin secretion rate and lower fasting glucagon concentration in healthy womenDiabetologia55123228323720122293596210.1007/s00125-012-2701-3CalissendorffJBrismarKRojdmarkSIs decreased leptin secretion after alcohol ingestion catecholamine-mediated?Alcohol and Alcoholism39428128620041520815710.1093/alcalc/agh054CampfieldLASmithFJBurnPThe OB protein (leptin) pathway—a link between adipose tissue mass and central neural networksHormone and Metabolic Research28126196321996901373110.1055/s-2007-979867CoelhoMOliveiraTFernandesRBiochemistry of adipose tissue: An endocrine organArchives of Medical Science9219120020132367142810.5114/aoms.2013.33181PMC3648822ConigraveKMHuBFCamargoCAJrA prospective study of drinking patterns in relation to risk of type 2 diabetes among menDiabetes50102390239520011157442410.2337/diabetes.50.10.2390De JesusLACarvalhoSDRibeiroMOThe type 2 iodothyronine deiodinase is essential for adaptive thermogenesis in brown adipose tissueJournal of Clinical Investigation10891379138520011169658310.1172/JCI13803PMC209445de MenezesRFBergmannAThulerLCAlcohol consumption and risk of cancer: A systematic literature reviewAsian Pacific Journal of Cancer Prevention1494965497220132417576010.7314/apjcp.2013.14.9.4965DembeleKNguyenKHHernandezTANyombaBLEffects of ethanol on pancreatic beta-cell death: Interaction with glucose and fatty acidsCell Biology and Toxicology25214115220091833071310.1007/s10565-008-9067-9DongHKumarMZhangYGamma-aminobutyric acid up- and downregulates insulin secretion from beta cells in concert with changes in glucose concentrationDiabetologia49469770520061644705810.1007/s00125-005-0123-1FengLHanBWangRThe frequency of daily ethanol consumption influences the effect of ethanol on insulin sensitivity in rats fed a high-fat dietBritish Journal of Nutrition107685085720122189298210.1017/S0007114511003722HaasSLYeWLöhrJMAlcohol consumption and digestive tract cancerCurrent Opinion in Clinical Nutrition and Metabolic Care15545746720122279757010.1097/MCO.0b013e3283566699HodgeAMDowseGKCollinsVRZimmetPZAbnormal glucose tolerance and alcohol consumption in three populations at high risk of non-insulin-dependent diabetes mellitusAmerican Journal of Epidemiology13721781891993845212210.1093/oxfordjournals.aje.a116658HolbrookTLBarrett-ConnorEWingardDLA prospective population-based study of alcohol use and non-insulin-dependent diabetes mellitusAmerican Journal of Epidemiology13259029091990223990510.1093/oxfordjournals.aje.a115733HotamisligilGSArnerPCaroJFIncreased adipose tissue expression of tumor necrosis factor-alpha in human obesity and insulin resistanceJournal of Clinical Investigation955240924151995773820510.1172/JCI117936PMC295872KangLSebastianBMPritchardMTChronic ethanol-induced insulin resistance is associated with macrophage infiltration into adipose tissue and altered expression of adipocytokinesAlcoholism: Clinical and Experimental Research3191581158820071762499410.1111/j.1530-0277.2007.00452.xKasztelan-SzczerbinskaBSurdackaASlomkaMAssociation of serum adiponectin, leptin, and resistin concentrations with the severity of liver dysfunction and the disease complications in alcoholic liver diseaseMediators of Inflammation201314852620132425994710.1155/2013/148526PMC3821915KimJYSongEHLeeHJChronic ethanol consumption-induced pancreatic β-cell dysfunction and apoptosis through glucokinase nitration and its down-regulationJournal of Biological Chemistry28548372513726220102085589310.1074/jbc.M110.142315PMC2988331KoppesLLDekkerJMHendriksHFModerate alcohol consumption lowers the risk of type 2 diabetes: A meta-analysis of prospective observational studiesDiabetes Care28371972520051573521710.2337/diacare.28.3.719LinHZYangSQZeldinGDiehlAMChronic ethanol consumption induces the production of tumor necrosis factor-alpha and related cytokines in liver and adipose tissueAlcoholism: Clinical and Experimental Research225 Suppl231S237S1998972764210.1097/00000374-199805001-00004MaedaNShimomuraIKishidaHDiet-induced insulin resistance in mice lacking adiponectin/ACRP30Nature Medicine8773173720021206828910.1038/nm724NicolasJMFernandez-SolaJFatjoFIncreased circulating leptin levels in chronic alcoholismAlcoholism: Clinical and Experimental Research2518388200111198718ObradovicTMeadowsGGChronic ethanol consumption increases plasma leptin levels and alters leptin receptors in the hypothalamus and the perigonadal fat of C57BL/6 miceAlcoholism: Clinical and Experimental Research262255262200211964566OuchiNKiharaSAritaYAdiponectin, an adipocyte-derived plasma protein, inhibits endothelial NF-kappaB signaling through a cAMP-dependent pathwayCirculation102111296130120001098254610.1161/01.cir.102.11.1296PattoRJRussoEKBorgesDRNevesMMThe enteroinsular axis and endocrine pancreatic function in chronic alcohol consumers: Evidence for early beta-cell hypofunctionMount Sinai Journal of Medicine60431732019938232378RogersCQAjmoJMYouMAdiponectin and alcoholic fatty liver diseaseIUBMB Life601279079720081870965010.1002/iub.124SierksmaAPatelHOuchiNEffect of moderate alcohol consumption on adiponectin, tumor necrosis factor-alpha, and insulin sensitivityDiabetes Care27118418920041469398710.2337/diacare.27.1.184StrbakVBenickyJMachoLFour-week ethanol intake decreases food intake and body weight but does not affect plasma leptin, corticosterone, and insulin levels in pubertal ratsMetabolism4710126912731998978163310.1016/s0026-0495(98)90335-3ThamerCHaapMFritscheARelationship between moderate alcohol consumption and adiponectin and insulin sensitivity in a large heterogeneous populationDiabetes Care275124020041511156210.2337/diacare.27.5.1240Tomie FuruyaDBinsackROnishiMELow ethanol consumption induces enhancement of insulin sensitivity in liver of normal ratsLife Sciences77151813182420051591365810.1016/j.lfs.2004.12.046VillarroyaJCereijoRVillarroyaFAn endocrine role for brown adipose tissue?American Journal of Physiology: Endocrinology and Metabolism3055E567E57220132383952410.1152/ajpendo.00250.2013WangSLuoYFengAEthanol induced impairment of glucose metabolism involves alterations of GABAergic signaling in pancreatic β-cellsToxicology326445220142545626510.1016/j.tox.2014.10.005WeiMGibbonsLWMitchellTLAlcohol intake and incidence of type 2 diabetes in menDiabetes Care231182220001085796210.2337/diacare.23.1.18WelschTKleeffJSeitzHKUpdate on pancreatic cancer and alcohol-associated riskJournal of Gastroenterology and Hepatology21Suppl3S69S751695867710.1111/j.1440-1746.2006.04574.xXuAWangYKeshawHThe fat-derived hormone adiponectin alleviates alcoholic and nonalcoholic fatty liver diseases in miceJournal of Clinical Investigation11219110020031284006310.1172/JCI17797PMC162288YokotaTOritaniKTakahashiIAdiponectin, a new member of the family of soluble defense collagenes, negatively regulates the growth of myelomonocytic progenitors and the functions of macrophagesBlood96517231732200010961870ZhangYProencaRMaffeiMPositional cloning of the mouse obese gene and its human homologueNature37265054254321994798423610.1038/372425a0

Similar findings have been obtained in animal studies. In a rat model of binge ethanol exposure, intraperitoneal injection of one dose of ethanol resulted in a significant decline of GH serum levels at 0.5, 1.5, and 3 hours compared with saline-injected control rats (Emanuele et al. 1992). In a model of chronic alcohol exposure, rats receiving 5 percent ethanol in a liquid diet for 4.5 months showed a significant decrease in circulating IGF-1 levels (Sonntag and Boyd 1988). Similarly, chronic 6-day administration of 5 percent ethanol to awake rats resulted in a 75 to 90 percent decrease in spontaneous GH secretion (Soszynski and Frohman 1992). In addition, ethanol treatment was associated with significant declines in IGF-I serum levels and GHRH mRNA levels, whereas somatostatin or GH mRNA levels did not change (Soszynski and Frohman 1992). These results suggest that chronic ethanol affects GH secretion primarily at the hypothalamic level where it induces impairments in GHRH gene expression. However, the responsiveness of the anterior pituitary to a GHRH challenge was the same in both saline- and ethanol-injected animals (Dees et al. 1988).

As mentioned earlier, the GH/IGF-1 pathway regulates carbohydrate and lipid metabolism. Recent studies have suggested that alcohol-induced changes in the circulating levels of IGF-1 and GH might contribute to the alcohol-mediated development of glucose intolerance and type 2 diabetes. In a rat model of type 2 diabetes (i.e., the type-2 diabetic Otsuka Long-Evans Tokushima Fatty rat model), alcohol administration significantly decreased IGF-1 serum levels and increased GH serum levels compared with nondiabetic control rats (Kim et al. 2013). These effects on IGF-1 and GH might contribute to the alcohol-mediated exacerbation of type 2 diabetes in the rats.

## Alcohol and the HPP Axis

The HPP axis includes two neuropeptides—AVP and oxytocin—both of which are produced by cells whose cell bodies are located in the hypothalamus but that extend to the posterior pituitary, where they release their hormones. AVP can be produced by two types of cells (i.e., magnocellular and parvocellular cells). Magnocellular neurosecretory cells produce the AVP that is found in peripheral blood. This AVP is secreted in response to osmotic stimuli and is involved in regulating the concentration of dissolved molecules (i.e., osmolality) in the body fluids by retaining water in the body and constricting blood vessels (Iovino et al. 2012; Verbalis 1993). In contrast, AVP produced by the parvocellular system is secreted following psychological stress and is involved in potentiating the action of CRF on ACTH release (Romero and Sapolsky 1996). Some AVP also may be released directly into the brain, and accumulating evidence suggests it plays an important role in social behavior, sexual motivation and pair bonding, and maternal responses to stress (Insel 2010). In the context of chronic alcohol use, AVP is involved in the disturbed water balance observed in actively drinking people with AUD and during acute withdrawal (Döring et al. 2003; Ehrenreich et al. 1997). AVP also may affect cognitive function, because treatment of alcoholic patients with memory deficits by using AVP analogs resulted in improved cognitive performance (Laczi 1987). Finally, studies in rodents have suggested that AVP may play a role in the development and maintenance of alcohol tolerance (Hoffman 1994).

Like AVP, oxytocin is produced by both magnocellular and parvocellular neurons of the hypothalamus. It functions both as a peripheral hormone and as a signaling molecule in the central nervous system (Buijs 1983). In its role as a peripheral hormone, oxytocin is released into the circulation from the posterior pituitary, enhancing uterine contractions during labor and, together with prolactin, enhancing milk release during lactation (Leng et al. 2015). Maternal alcohol use before or during lactation can interfere with the proper function of both prolactin and oxytocin (Heil and Subramanian 1998). In the central nervous system, oxytocin is released by a variety of neurons. Some of these are neurons whose cell bodies are in the hypothalamus and that extend to limbic and forebrain areas, where they release oxytocin from their terminals. Other oxytocin-releasing neurons are located outside the hypothalamus, in the amygdala and bed nucleus of the stria terminalis (Ross and Young 2009). Oxytocin may be a major contributor to alcohol tolerance and dependence (Hoffman and Tabakoff 1981; McGregor et al. 2012). Moreover, recent studies have demonstrated that peripheral administration of oxytocin can reduce ethanol consumption in rats (MacFadyen et al. 2016) and that intranasal oxytocin administration blocks alcohol withdrawal in humans (Pedersen et al. 2013).

## Conclusion

Alcohol’s deleterious effects on the endocrine system have far-reaching consequences that can result in serious physiological and behavioral disorders. Alcohol abuse not only causes hormonal disturbances, but because these disturbances permeate every organ and tissue in the body, can result in various debilitating disorders, such as stress intolerance, disturbed water balance and body osmolality, reproductive dysfunction, thyroid problems, immune abnormalities, diabetes, cardiovascular disease, cancer, and psychological and behavioral disorders. The different components of the endocrine system, particularly the HPA axis, HPG axis, HPT axis, GH/IGF-1 axis, and HPP systems, normally communicate with each other as well as with the nervous and immune systems in response to external environmental cues and help maintain homeostasis and health. These coordinated bidirectional interactions rely on the production and release of chemical messengers, such as neurotransmitters, hormones, and cytokines, that mediate the communications between the different systems. Alcohol abuse disrupts the release of these chemical signals and negatively affects the communication pathways. A better understanding of the mechanisms involved in alcohol’s effects on the bidirectional interactions between the HPA, HPG, HPT, and GH/IGF-1 axes; the HPP system; and the immune system will help pave the way for the development of effective therapeutic tools for AUD.

## Figures and Tables

**Table:** Summary of Important Hormones, Their Sites of Production, the Hormone System They Belong to, and Their Main Functions or Target Organs

Site of Production	Hormone	Hormone System	Main Function or Target Organ
**Hypothalamus**	Corticotropin-releasing factor	Hypothalamic–pituitary–adrenal axis	Anterior pituitary gland
	Luteinizing hormone–releasing hormone	Hypothalamic–pituitary–gonadal axis	Anterior pituitary gland
	Thyrotropin-releasing hormone	Hypothalamic–pituitary–thyroid axis	Anterior pituitary gland
	Growth hormone–releasing hormone	Growth hormone/insulin-like growth factor-1	Anterior pituitary gland
	Somatostatin	Growth hormone/insulin-like growth factor-1, Hypothalamic–pituitary–thyroid axis	Anterior pituitary gland
	Dopamine	Prolactin	Anterior pituitary gland
**Anterior Pituitary Gland**	Adrenocorticotropic hormone	Hypothalamic–pituitary–adrenal axis	Adrenal cortex
	Thyroid-stimulating hormone	Hypothalamic–pituitary–thyroid axis	Thyroid
	Follicle-stimulating hormone	Hypothalamic–pituitary–gonadal axis	Gonads (ovaries, testes)
	Luteinizing hormone	Hypothalamic–pituitary–gonadal axis	Gonads (ovaries, testes)
	Growth hormone	Growth hormone/insulin-like growth factor-1	Growth and repair of all cells
	Prolactin	Prolactin	Breast
**Hypothalamus/Posterior Pituitary Gland**	Arginine vasopressin	Hypothalamic–pituitary–adrenal axis	Blood vessels and kidney
	Oxytocin	Oxytocin	Uterus, mammary glands, male reproductive organs
**Adrenal Glands**	Glucocorticoids (cortisol, corticosterone)	Hypothalamic–pituitary–adrenal axis	Body stress, metabolism, glucose maintenance
**Ovary (Follicle)**	Estrogen (estrone, estradiol, estriol)	Hypothalamic–pituitary–gonadal axis	Female reproductive glands and tissues, bones, heart
**Ovary (Corpus Luteum)**	Progesterone	Hypothalamic–pituitary–gonadal axis	Maintenance of pregnancy and preparation of breast tissue
**Testes**	Testosterone	Hypothalamic–pituitary–gonadal axis	Masculinity, sperm production, bone
**Thyroid**	Thyroxine (T4) Triiodothyronine (T3)	Hypothalamic–pituitary–thyroid axis	Heart rate, temperature, metabolism
**Pancreas**	Insulin Glucagon	Pancreas Pancreas	Lower blood sugar Increase blood sugar

## Footnotes

1 Norepinephrine also is released from postganglionic neurons of the sympathetic nervous system.

2 Note that BEP also acts as an endogenous opioid peptide with pain-relieving (i.e., antinociceptive) effects.

## Glossary

Anabolic: Pertaining to the metabolic processes by which organisms convert substances into other components the body needs
Apoptosis: Specific pattern of reactions resulting in the death of single cells; also referred to as programmed cell death
Autocrine: A mode of hormone action in which a hormone binds to receptors on, and affects the functions of, the cell type that produced it
Autonomic Nervous System: Part of the nervous system that connects the central nervous system to the organs and controls involuntary bodily functions, such as respiration and digestion
C-Peptide: Part of the precursor molecule of insulin that gets excised during the final processing of the insulin molecule; has no physiologic activity
Epididymal: Pertaining to the epididymis—the elongated, cordlike structure along the rear of the testis that provides for storage, transit, and maturation of sperm
Epigenetic: Altering the activity of genes without changing their DNA sequences (e.g., through chemical modification of the DNA or the histone proteins around which the DNA is coiled)
Fecundability: The probability that a woman becomes pregnant in a certain period of time
Glycogen: A large, highly branched molecule consisting of chains of glucose molecules; constitutes the major carbohydrate reserve of animals and is stored primarily in liver and muscle
Insulin Resistance: Impairment of the normal physiological response to insulin that may be the result of a variety of abnormalities; occurs in diabetes mellitus
Paracrine: A mode of hormone action in which a hormone binds to receptors on, and affects the functions of, nearby cells of a different type from the cell type that produced it
Parasympathetic Nervous System: Part of the *autonomic nervous system* that operates to help the body conserve energy and resources in a relaxed state
Promoter: Segment of DNA usually in front of a gene that acts as a controlling element in the expression of that gene
Reactive Oxygen Species: Biologically active, partially reduced derivatives of molecular oxygen that are produced by normal metabolic processes and which can damage the cells or their components
Sympathetic Nervous System: Part of the *autonomic nervous system* that stimulates organs and blood vessels to help the body react to stressful situations
Total Integrated Response: A measure of the area under the curve of the insulin or glucose response to an oral glucose challenge used to determine *insulin resistance*
